# The highly efficient photocatalytic and light harvesting property of Ag-TiO_2_ with negative nano-holes structure inspired from cicada wings

**DOI:** 10.1038/s41598-017-17479-8

**Published:** 2017-12-08

**Authors:** Imran Zada, Wang Zhang, Wangshu Zheng, Yuying Zhu, Zhijian Zhang, Jianzhong Zhang, Muhammad Imtiaz, Waseem Abbas, Di Zhang

**Affiliations:** 1 State Key Laboratory of Metal Matrix Composites, Shanghai Jiao Tong University, 800 Dongchuan Road, Shanghai, 200240 P.R. China; 2 Jushi Fiberglass Research Institute, Jushi Group Co., Ltd. 669 Wenhua Road (South), Tongxiang Economic Development Zone, Tongxiang City, Zhejiang Province 314500 P.R. China

## Abstract

The negative replica of biomorphic TiO_2_ with nano-holes structure has been effectively fabricated directly from nano-nipple arrays structure of cicada wings by using a simple, low-cost and highly effective *sol-gel* ultrasonic method. The nano-holes array structure was well maintained after calcination in air at 500 °C. The Ag nanoparticles (10 nm–25 nm) were homogeneously decorated on the surface and to the side wall of nano-holes structure. It was observed that the biomorphic Ag-TiO_2_ showed remarkable photocatalytic activity by degradation of methyl blue **(**MB) under UV-vis light irradiation. The biomorphic Ag-TiO_2_ with nano-holes structure showed superior photocatalytic activity compared to the biomorphic TiO_2_ and commercial Degussa P25. This high-performance photocatalytic activity of the biomorphic Ag-TiO_2_ may be attributed to the nano-holes structure, localized surface plasmon resonance (LSPR) property of the Ag nanoparticles, and enhanced electron-hole separation. Moreover, the biomorphic Ag-TiO_2_ showed more absorption capability in the visible wavelength range. This work provides a new insight to design such a structure which may lead to a range of novel applications.

## Introduction

Photolysis of organic containments has paid a great attention in environmental cleaning and water purification^1–5^. TiO_2_ has been extensively used as a photocatalytic material due to its significant photostability, environmentally friendliness, high chemical stability, non-toxicity and low-cost^6^. It has potential applications in decomposition of organic pollutants^7,8^ water splitting^9^ and photo energy conversion^10^, etc. However, owing to their wide band gap (i.e., 3.2 eV for anatase and 3.0 eV for rutile), TiO_2_ absorb mainly UV light which is only 4% of the entire solar spectrum on earth^11^. Therefore, several techniques have been applied to exploit most of the energy spectrum (visible region) which is about 40% of the total energy^12^. These techniques involved doping of TiO_2_ with metallic or nonmetallic elements^11^, sensitizing TiO_2_ with a second photoactive component such as ruthenium complex^13^, quantum dots^14^, organic dyes^15^, and narrow bandgap semiconductors^16^. Even though the above-mentioned methods partially enhance the photocatalytic property of TiO_2_, yet still it limits their efficiency due to the thermal instability, lower redox potential of the photo-generated electrons and reduced electron-hole separation^11^. Moreover, it has been also reported that different micro-nano structures significantly enhances the light harvesting and photocatalytic property of TiO_2_ such as nanotube arrays^17^, ordered mesoporous structure^18^, micro-hole arrays^19^, inverse opal structures^20^. To improve light absorption, these structures allow the incident light through cavities and reduces the optical loss due to the multiple light scattering and reflection. However, it’s very challenging for new technology to design and produce such effective nano-architectures with high symmetry, while one can easily find these architectures in nature.

In fact, nature provides us an astonishing variety of structures from biological system such as periodic structure of butterfly wings^21,22^, cicada wings^23,24^, green leaves^25,26^, the sea-mouse spines^27^ and the insects compound eyes^28^. Among these biological prototypes, cicada wing is one of the most promising template due to its highly ordered hexagonal nano-nipple arrays structure, which plays an important role in reducing light reflection over a broad range of visible wavelength^24^. Chitin, a crystalline polymer with a high Young’s modulus of 7–9 GPa and high molecular weight, is the most important component of the cicada wing, and has a key role in preserving the original nanostructure of the wing surface^29^. Therefore, on large scale existing of these two dimensional nano-nipple arrays structure of cicada wing was found to be a most useful natural biotemplate during the replication process. Many researchers have made different approaches to replicate the negative replica of cicada wings nano-structure such as metal deposition^30^, nanoimprint lithography^31,32^, and low-surface-energy resin^33^. Guoyong *et al*. prepared the negative replica of Au directly from cicada template and then used it as a mold to fabricate the positive replica of PMMA (polymethyl methacrylate)^30^. Xinyue and Guoming *et al*. also produced the negative polymeric replica from cicada wings which shows better optical performance^32,33^. However, these synthetic methods are expensive and the low thermal stability of polymers limits their applications for outdoor uses. Therefore, in this study we used a simple and inexpensive *sol-gel* ultrasonic method to replicate one of the most interesting semiconductor titanium dioxide (TiO_2_) with nano-holes array structure which was further decorated with Ag nanoparticles to exhibit the better performance of photocatalytic activity under the UV-Vis light source.

Nowadays, noble metal nanoparticles have been used to enhance the photocatalytic performance of TiO_2_ under the visible light irradiation. These noble nanoparticles strongly connect with the light due to their excitation of surface plasmon resonance (SPR) and enhanced light harvesting property of nanocomposite^34^. The size, shape, composition, and the nature of the metallic nanostructure strongly affect the intensity and frequency of SPR which make it possible to design plasmonic nanostructures that interact with whole solar spectrum^35,36^. Moreover, the slow photon effect could also significantly enhance the photocatalytic performance due to the strong interaction of light with matter. The slow photon effect was raised from the reduced group velocity which increase the optical path of light wave and improves the light harvesting capability of the solar spectrum^11^. The inverse opal structure of wideband semiconductor TiO_2_ and ZnO showed better performance of photocatalytic activity owing to the slow photon effect^37,38^. Among the noble metals, Ag has attracted special attention due to inexpensive, non-toxic and easy preparation methods compared to the other noble metals. Therefore, the decoration of Ag nanoparticles on the surface of TiO_2_ composite has become a great interest. The Ag nanoparticles on the surface of TiO_2_ has been used to effectively maximize the photocatalytic reaction. The Fermi level of these Ag nanoparticles is below the conduction band of TiO_2_ which can play a role of electron scavenging centers to separate the electron-hole pair, and drastically increase the photocatalytic performance of TiO_2_^39^. For example, different research works have shown the photocatalytic activity of Ag-TiO_2_ nanocomposite such as thin films^40^, nanowires^41^, nanorods^42^ and Ag modified TiO_2_ nanoparticles^43^. However, there is still some considerable difficulties due to the complicated experimental procedures and complex structural fabrication. The Ag nanoparticle was also deposited on surface of TiO_2_ photocatalysts via photochemical reduction through UV light irradiation^44^. This method was easy and inexpensive, but the weak bonding force between Ag and TiO_2_ and aggregation of Ag on surface of TiO_2_ decreased the light absorption as well as charge transfer rate. Besides, the photocatalytic performance of Ag-TiO_2_ was mostly studied only under the UV light illumination. Therefore, it was vital to fabricate Ag-TiO_2_ with strong bonding between Ag nanoparticles and TiO_2_ and to show a significant photocatalytic activity under UV-Vis light irradiation.

Herein, we fabricated biomorphic Ag-TiO_2_ with nano-holes array structure directly from cicada wings nanostructure after high calcination using a simple and inexpensive *sol-gel* ultrasonic method. Owing to the strong connection of SPR with *in situ* biomorphic TiO_2_, Ag nanoparticles effectively degrade MB under UV-Vis light irradiation.

## Results and Discussion

**Figure 1:**
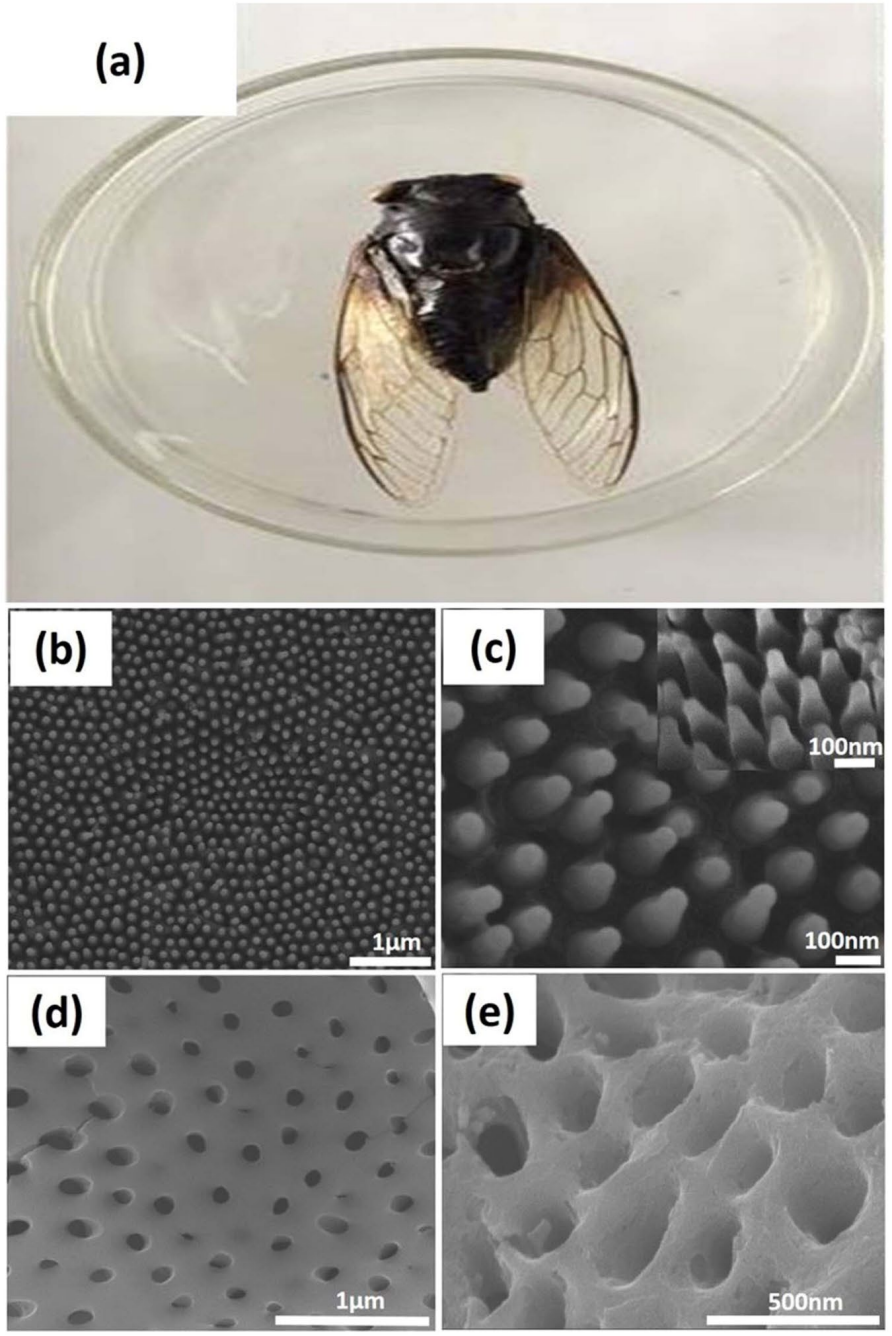
(**a**) A photograph of the black cicada wing (**b**) Low magnified SEM image of the highly ordered nano-nipple arrays structure (**c**) Highly magnified side view SEM image of nano-nipple arrays structure. (**d**) Top view SEM image of negative replica of the biomorphic TiO_2_. (**e**) Highly magnified side view SEM image of the biomorphic TiO_2_.

In this work, black cicada was selected as the biological prototype due to its nano-nipple arrays structure on the surface of wing. It can be seen that the cicada wings are mostly transparent excluding some supporting veins and edge regions with cross-sectional thicknesses of 60−150 μm^45^. Figure 1a shows a photograph of the black cicada wing. The transparent wing section consist of nano-nipple arrays structure as shown by SEM images in Fig. 1b,c. It can be seen that the nano-nipple arrays on entire wing surface were arranged in highly order. The average separation distance between center to center (160 nm), basal diameter (140 nm), and height (210 nm) of the nano- nipple arrays are shown in Fig. 1c and its inset. It has been proved that these kind of subwavelength structures not only favors to the excellent antireflection property but also shows low adhesion, superhydrophobicity and self-cleaning properties^46^. Figure 1d,e shows the negative replica of biomorphic TiO_2_ with nano-holes structure after high calcination in air at 500 °C. The nano-holes arrays were uniformly distributed after complete removal of biotemplate. The biomorphic TiO_2_ effectively replicated the negative structure of cicada wing and maintained both the size and shape. The average structural parameters of the negative nano-holes arrays were found to be 220 nm in depth, 120 nm in diameter and 275 nm in central distance. These values indicate that a little distortion has been occurred after direct replication of nanostructures of cicada wing at high calcination.

As it is well known that the wing membrane of cicada wing is mainly composed of chitin, protein and wax component. Therefore, the wings were pretreated with 8% NaOH to remove inorganic salts from the surface of wings. After this modification, the precursor sol can easily flow into the internal structure of the wing and the final obtained replica well preserve the nanostructure of the original wings. The possible detail mechanism for the replication process of TiO_2_ is given below,

The reaction between TiCl_4_ and ethanol produces metallic chloroethoxide precursors [TiCl_4−x_ (OEt)], with x ≈ 2 as shown in equation (1)^47^:

1 $${{\rm{TiCl}}}_{4}+2{\rm{EtOH}}\to {{\rm{TiCl}}}_{2}{({\rm{OEt}})}_{2}+2{\rm{HCl}}$$

when water was added to the solution, the initial chloro-alkoxide complex TiCl_2_ (EtO)_2_ was modified through hydrolysis of the inorganic moieties which produce *in situ* ethanol and HCl as shown by equation (2). The maximum concentration of local HCl in the final solutions hampers inorganic polymerization and can extend inorganic polymerization from several minutes to days, and thus increase the stability of the sols:

2 $$\begin{array}{c}{{\rm{TiCl}}}_{2}{({\rm{OEt}})}_{2}\,\mathop{\longrightarrow }\limits^{{{\rm{mH}}}_{2}{\rm{O}}}\,{[{\rm{Ti}}{({\rm{OH}})}_{2}{({{\rm{H}}}_{2}{\rm{O}})}_{{\rm{n}}}({{\rm{Z}}}_{{\rm{x}}})]}^{(2-{\rm{x}})}+(2\,-\,{\rm{xHCl}})+(2\,-\,{\rm{xEtOH}})\\ {\rm{Z}}=\text{OH},\,{\rm{Cl}},\,{\rm{OEt}}\,{\rm{m}} > 4;\,{\rm{x}} < 2.\end{array}$$

Therefore, the hydrolysis and a small extent of condensation in these highly acidic condition make it possible to prepare the initial TiCl_4_/EtOH/H_2_O/template solutions^48^. The cicada wings surface with hydroxy groups can easily diffuse the gel inside nanostructures. Here, the HCl produced *in situ* can be eliminated by the evaporation process, allowing an extended inorganic condensation. When the reaction was agitated through ultrasound, the solutes homogeneously dispersed in the solution, catalyze the hydrolysis and condensation of the Titania precursor to TiO_2_. And thus, an intact sol particles layer is formed on the surface of wings after being taken out from precursor solution. Finally, the negative replica of biomorphic TiO_2_ with nano-holes array structure was produced after high calcination.

**Figure 2:**
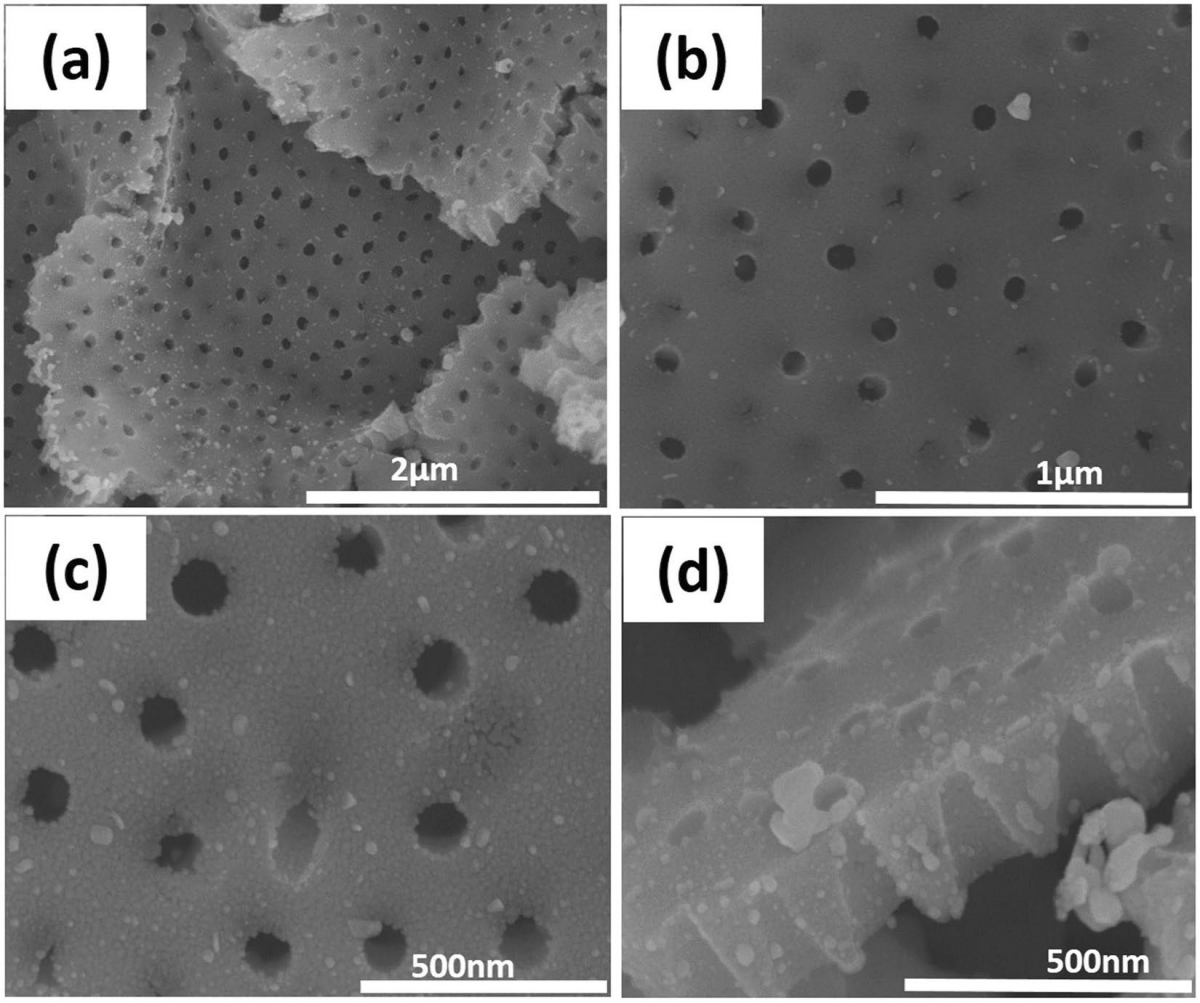
(**a**) Low magnified top view SEM image of biomorphic Ag-TiO_2_ with nano-holes array structure. (**b**) and (**c**) highly magnified top view SEM images of biomorphic Ag-TiO_2_ with nano-holes array structure. (**d**) Highly magnified side view SEM image of biomorphic Ag-TiO_2_.

Figure 2 shows the SEM images of the negative replica of biomorphic Ag-TiO_2_ with nano-holes array structure. From Fig. 2a, it can be clearly seen that the biomorphic Ag-TiO_2_ with nano-holes array structure were uniformly distributed and there were no obvious defects. The Ag nanoparticles with a size of 10–25 nm were uniformly dispersed on the surface of biomorphic TiO_2_ as shown in Fig. 2b,c. It was obvious that the Ag nanoparticles were not only deposited on the surface of biomorphic TiO_2_ but also to the side wall of the nano-holes (Fig. 2d). As such, there is no aggregation of Ag nanoparticles to block the nano-holes of the obtained biomorphic Ag-TiO_2_ samples. A small cluster shown in the SEM images (Fig. 2b,d) might be due to the some impurity. The average depth, diameter and center to center distance of the nano-holes array were 220 nm, 110 nm, 275 respectively, as shown in Fig. 2c,d. These parameters show that the negative replica of original cicada wing was inherited to a great extent after high calcination. A shrinkage of 21% was observed in the diameter of the nano-holes compared to the basal diameter of the original wing. On the other hand, deformation of 41% and 3.6% was observed in the size of central spacing and depth of nano-holes structure, respectively. The shrinkage and deformation happened due to the high calcination temperature and uneven coating. It has been previously shown that the nanostructure morphology can be affected under high calcination temperatures^23^.

**Figure 3:**
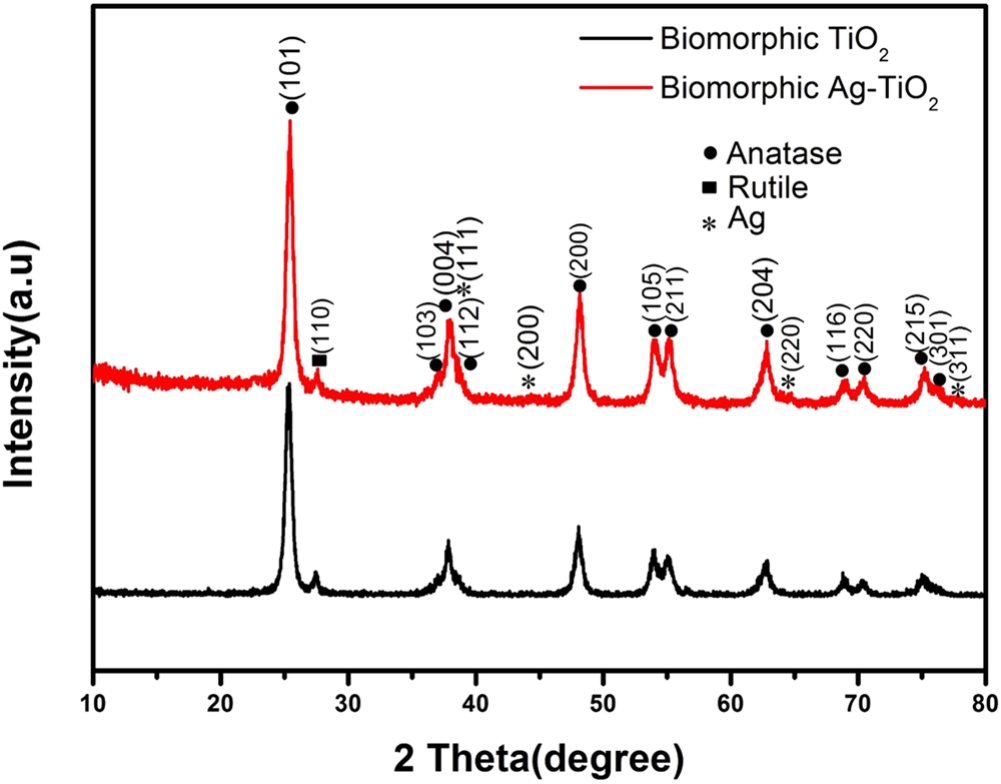
XRD patterns of the biomorphic TiO_2_ and biomorphic Ag-TiO_2_.

Figure 3 shows the XRD patterns of the biomorphic TiO_2_ and biomorphic Ag-TiO_2_. The diffraction peaks of anatase phase was centered at 2θ = 25.28, 37.80, 48.04, 53.89, 55.06, 62.68, 68.76, 70.30 and 75.02° which correspond to the (101), (004), (200), (105), (211), (204) (116), (220) and (215) planes. A small peak of rutile phase was also observed at 2θ = 27.44 corresponding to (110) plane due to the high calcination temperature. The diffraction peaks of Ag were not very obvious because the (111) plane of Ag at 2θ = 37.80 were overlapped to the diffraction peaks of biomorphic TiO_2_ (004) planes^49^. The diffraction peaks of biomorphic TiO_2_ were not disturbed by the deposition of Ag nanoparticles as shown in Fig. 3. This shows that the Ag elements did not enter into the crystal lattice of TiO_2_^50^.

**Figure 4:**
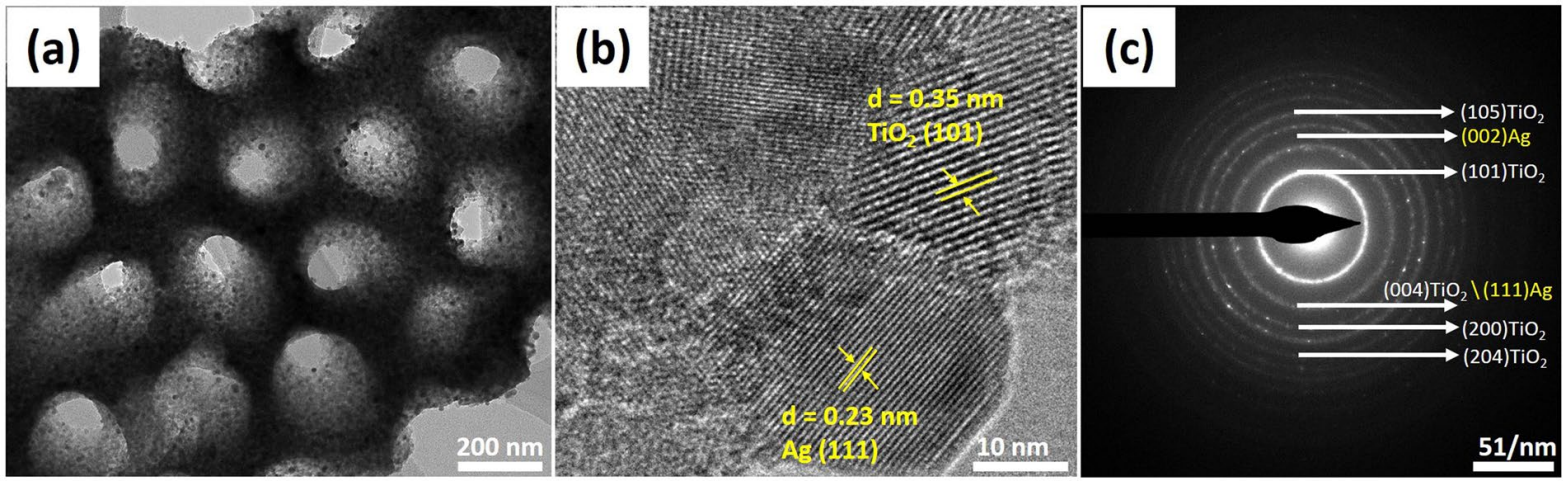
(**a**) TEM image of the biomorphic Ag-TiO_2_. (**b**) HRTM image of the biomorphic Ag-TiO_2_. (**c**) SAED pattern of the biomorphic Ag-TiO_2_.

The detail morphology and microstructure of the biomorphic Ag-TiO_2_ was further investigated by TEM. From Fig. 4a, it can be clearly seen that the negative replica of TiO_2_ with nano-holes structure was well maintained after high calcination. The Ag nanoparticles were homogeneously deposited on the surface and to the side wall of nano-holes structure of biomorphic TiO_2_, which is in well agreement to the SEM results. The high-resolution transmission electron microscopy (HRTEM) image of the biomorphic Ag-TiO_2_ was shown in Fig. 4b to investigate the heterojunctions between Ag and biomorphic TiO_2_. It was observed that the lattice fringe with a space of 0.35 nm corresponds to the (101) lattice plane of anatase TiO_2_, while the interlayer space of 0.23 belonging to the (111) cubic face plane of Ag. Figure 4c shows the selected area electron diffraction (SAED) pattern of biomorphic Ag-TiO_2_. The diffraction rings clearly present the mixed phase of anatase TiO_2_ and cubic Ag. The diffraction rings indexed with (111) and (002) faces corresponds to the metallic Ag.

**Figure 5:**
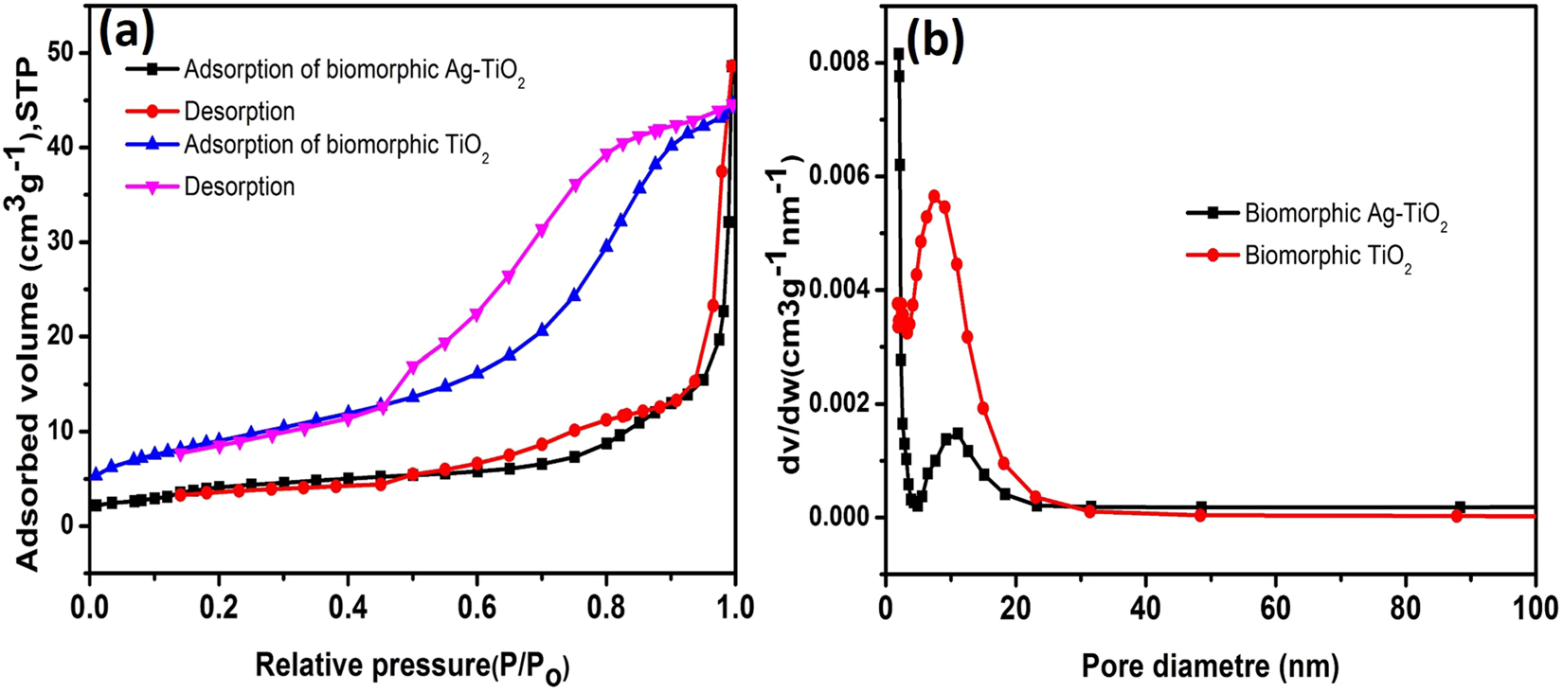
(**a**) N_2_ adsorption/desorption isotherm measurements of biomorphic TiO_2_ and Ag-biomorphic TiO_2_. (**b**) The pore size distribution of biomorphic TiO_2_ and biomorphic Ag-TiO_2_.

The surface area and porous structure of the biomorphic TiO_2_ and biomorphic Ag-TiO_2_ was determined by analysis of N_2_ adsorption/desorption isotherms as shown in Fig. 5. For both samples, a typical type IV isotherm with apparent hysteresis loops was detected in the relative pressure (P/P_o_) range of 0.45–1.0 (Fig. 5a). The upturn hysteresis loop confirms the existence of mesoporous structure of biomorphic TiO_2_ and biomorphic Ag-TiO_2_. The Brunauer-Emmet-Teller (BET) surface areas of biomorphic TiO_2_ and Ag-TiO_2_ were 33.0291 m²/g and 17.0921 m²/g, respectively. The lower surface area of the biomorphic Ag-TiO_2_ was due to the deposition of Ag nano-particles onto the surface of biomorphic TiO_2_. It has been previously shown that lower surface area of the three dimensional Ag-TiO_2_ nano-architecture was due to the deposition of Ag nano-particles which covered some mesopores and interstitial space of the TiO_2_ network^49^. The average pore diameters and the average pore volumes from the Barrett-Joyner-Halenda (BJH) desorption analysis for biomorphic TiO_2_ and Ag-TiO_2_ were 7.44 nm, 10.82 nm and 0.070231 cm³/g, 0.076065 cm³/g respectively. The pore size distribution curve in Fig. 5b confirm the mesopores.

**Figure 6:**
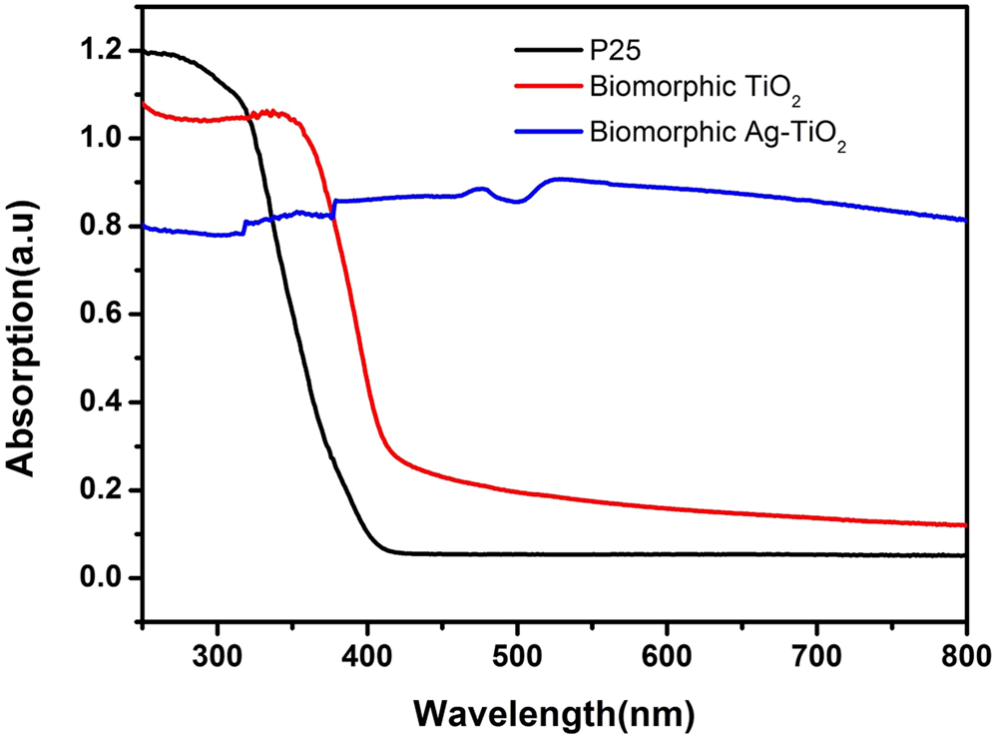
UV-vis absorption spectra of the biomorphic Ag-TiO_2_, biomorphic TiO_2_ and P25.

Figure 6 depicts the UV-vis absorption spectra of the P25, biomorphic TiO_2_ and biomorphic Ag-TiO_2_. It can be seen that the commercial P25 shows a photoresponse in the ultraviolet region only and there is a very low absorption in the visible wavelength range. But, the biomorphic TiO_2_ with nano-holes structure shows a bit more visible response compared to the commercial P25 powder. Whereas, the deposition of Ag nanoparticles onto the surface as well as to the side wall of the nano-holes structure prominently increases the visible light absorption compared to both biomorphic TiO_2_ and P25. It has been shown theoretically that the more absorption of light in the visible wavelength range owing to the SPR originated from the Ag nanoparticles in the Ag-TiO_2_ samples^51^. Therefore, it is quite possible that the biomorphic Ag-TiO_2_ with nano-holes structure may implicate excellent photocatalytic activity due to the high absorption in the visible wavelength range.

**Figure 7:**
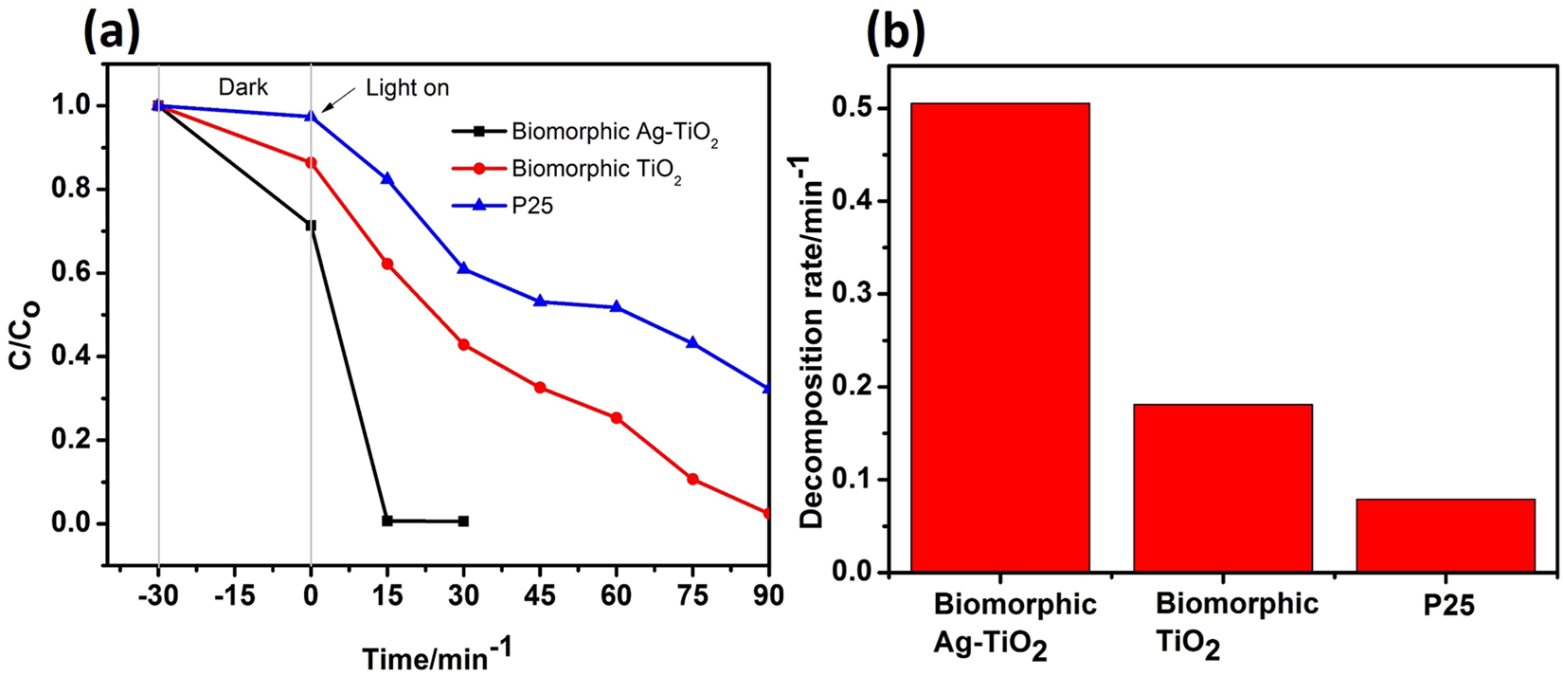
(**a**) The photocatalytic degradation of MB under UV-vis light irradiation. (**b**) The decomposition rate of biomorphic Ag-TiO_2_, biomorphic TiO_2_ and P25.

The photocatalytic activities of the samples were evaluated by decomposing MB dye under UV-vis light irradiation, as shown in Fig. 7. The photocatalytic performances of biomorphic Ag-TiO_2_, biomorphic TiO_2_ and nonporous commercial powder P25 were measured under the same condition as shown in Fig. 7a. Here, the rate of change of concentration (C/C_o_) was calculated with respect to time under UV-vis light irradiation (where C_o_ is the initial concentration of MB and C is the concentration at reaction time t). It can be clearly seen that the biomorphic Ag-TiO_2_ with nano-holes structure shows highest photocatalytic activity and completely degraded MB after 15 minutes. As it is apparent, the biomorphic TiO_2_ and P25 shows much lower photocatalytic activity than that of biomorphic Ag-TiO_2_. Moreover, the average decomposition rate of biomorphic Ag-TiO_2_ (0.50 min^−1^) was higher than that of biomorphic TiO_2_ (0.18 min^−1^) and P25 (0.07 min^−1^) as shown in Fig. 7b. The high-performance photocatalytic activity of biomorphic Ag-TiO_2_ may be attributed to the Schottky barriers which can be formed between Ag and biomorphic TiO_2_ with nano-holes and improves the electron-hole separation^52^. Another reason for the better photocatalytic performance of Ag-TiO_2_ with nano-holes structure is the enhanced LSPR intensity of Ag nanoparticles which in turn increase the absorption of light. The biomorphic TiO_2_ with nano-holes structure shows little photocatalysis of MB because pure TiO_2_ barely absorb visible light. The low performance of the P25 with high specific area (53 m^2^ g^−1^)^53^ was due to the absence of nano-holes structure compared to the biomorphic TiO_2_ and biomorphic Ag-TiO_2_. Therefore, the deposition of Ag nanoparticles on the surface of biomorphic TiO_2_ offer more active sites for the photocatalytic process.

## Conclusion

We have successfully fabricated negative replica of biomorphic Ag-TiO_2_ with nano-holes structure from cicada wings by using a simple and low cost sol-gel ultrasonic method. The biomorphic Ag-TiO_2_ effectively inherited the negative structure of cicada wing after high calcination at 500 °C. The homogeneous dispersion of Ag nanoparticles onto the surface and inside the nano-holes structure of TiO_2_ enhanced the photocatalytic performance by degradation of MB in the UV-vis region, which can be attributed to the LSPR property of Ag nanoparticles and enhanced electron-hole separation. The biomorphic Ag-TiO_2_ showed higher photocatalytic activity compared to the biomorphic TiO_2_ and P25. Therefore, this simple synthetic method is expected to open up fabrication strategies of such nano-hole structure materials which can find important applications in environmental and energy technologies.

## Experimental

### Synthesis of biomorphic TiO_2_

**Figure 8:**
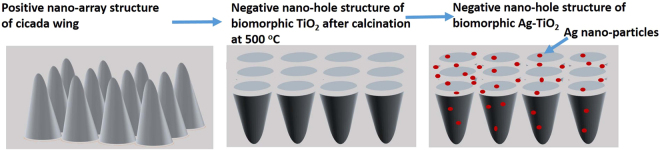
The schematic illustration of biomorphic TiO_2_ and biomorphic Ag-TiO_2_ with nano-holes structure.

Cicadas (Cryptotympana atrata Fabricius) were obtained from Shanghai Natural Wild-Insect Kingdom Co., Ltd. Analytical grade reagent, NaOH, AgNO_3_, NaBH_4_, absolute ethanol, titanium chloride(TiCl_4_), surfactant (TritonX-100) were provided by Shanghai chemical company. The cicada wings were firstly cleaned by water and ethanol three times each, and then dried in air. The cleaned wing were then pretreated by 8% NaOH solution and kept in water kettle for 3 hour at 40 °C. To prepare the precursor solution, firstly the 11 ml of deionized water was added with 35 ml of ethanol. After that 1 ml of TiCl_4_ was slowly added to the ethanol-water solution and keep at stirred for 1 h, while adding TritonX-100 dropwise in the precursor solution. The pretreated wings were then immersed carefully into the precursor solution and sonicated at room temperature for 2.5 h by high-intensity ultrasonic irradiation (20 kHz, 100 W cm^−2^). After ultrasonication, these wings were kept in the precursor solution for 5 h to solidify it, and then taken out, cleaned with ethanol and dried in air at room temperature. Finally, the wings were put on the glass slides and calcined at 500 °C for 2 h in air to eradicate the organic template, leaving behind biomorphic TiO_2_ with nano-holes structure. The schematic illustration for this synthetic process is shown in Fig. 8.

### Synthesis of biomorphic Ag-TiO_2_

The 55 mg of biomorphic TiO_2_ was added to the 30 ml of AgNO_3_ (20 mM) solution and vigorously mixed on magnetic stirrer for 1 h. After that, the precipitation was separated from the solution and washed with ethanol three times. The obtained precipitation was then reacted with 200 NaBH_4_ solution, followed by a filtration and dried in air for 60 °C to get the biomorphic Ag-TiO_2_.

### Characterization

The surface morphology of cicada wing, biomorphic TiO_2_ and biomorphic Ag-TiO_2_ were characterized by scanning electron microscopy (SEM; JSM-6700F, JEOL, Japan). X-ray diffraction patterns (XRD; CuKα, Bruker-AXS) with λ = 0.15406 nm of the samples were recorded from 10° to 80°. Transmission electron microscopy (TEM) images, high-resolution transmission electron microscopy (HRTEM) images and selected area electron diffraction (SAED) images were obtained on a JEOL JEM-2100F TEM. The specific surface area and pore size distribution of the samples were measured on a Micromeritics ASAP 2020 at 77 k. In the spectral range of 200 to 800 nm, the absorption spectra of the samples were measured on Varian Cary UV-vis-NIR spectrophotometer.

### Photocatalytic degradation of MB

The photocatalytic degradation of MB was performed under the UV-vis light irradiation (Xenon lamp, PLS-SXE 300/300UV, 10A). The photocatalytic reaction was maintained at room temperature by using fixed cooling fan in the light source to avoid any thermal catalytic effect. 30 mg of the as-prepared samples were mixed in 30 ml of MB aqueous solution (30 mg/l) and kept on magnetic stirring for 30 min in the dark to achieve the adsorption-desorption equilibrium among water, MB and photocatalyst. After equilibrium, the reaction system was exposed to the UV-vis light to assess the photocatalytic degradation. After each 15 min, 3 ml of solution was taken out from the reaction system and centrifuged to get clear liquid. The absorption spectra of the supernatants were recorded on 25 lambda UV-vis spectrometer.
